# Coding “What” and “When” in the Archer Fish Retina

**DOI:** 10.1371/journal.pcbi.1000977

**Published:** 2010-11-04

**Authors:** Genadiy Vasserman, Maoz Shamir, Avi Ben Simon, Ronen Segev

**Affiliations:** 1 Department of Life Sciences, Ben-Gurion University of the Negev, Beer-Sheva, Israel; 2 Zlotowski Center for Neuroscience, Ben-Gurion University of the Negev, Beer-Sheva, Israel; 3 Department of Physiology and Neurobiology, Ben-Gurion University of the Negev, Beer-Sheva, Israel; New York University, United States of America

## Abstract

Traditionally, the information content of the neural response is quantified using statistics of the responses relative to stimulus onset time with the assumption that the brain uses onset time to infer stimulus identity. However, stimulus onset time must also be estimated by the brain, making the utility of such an approach questionable. How can stimulus onset be estimated from the neural responses with sufficient accuracy to ensure reliable stimulus identification? We address this question using the framework of colour coding by the archer fish retinal ganglion cell. We found that stimulus identity, “what”, can be estimated from the responses of best single cells with an accuracy comparable to that of the animal's psychophysical estimation. However, to extract this information, an accurate estimation of stimulus onset is essential. We show that stimulus onset time, “when”, can be estimated using a linear-nonlinear readout mechanism that requires the response of a population of 100 cells. Thus, stimulus onset time can be estimated using a relatively simple readout. However, large nerve cell populations are required to achieve sufficient accuracy.

**Authors Summary**

In our interaction with the environment we are flooded with a stream of numerous objects and events. Our brain needs to understand the nature of these complex and rich stimuli in order to react. Research has shown ways in which a ‘what’ stimulus was presented can be encoded by the neural responses. However, to understand ‘what was the nature of the stimulus’ the brain needs to know ‘when’ the stimulus was presented. Here, we investigated how the onset of visual stimulus can be signalled by the retina to higher brain regions. We used archer fish as a framework to test the notion that the answer to the question of ‘when’ something has been presented lies within the larger cell population, whereas the answer to the question of ‘what’ has been presented may be found at the single-neuron level. The utility of the archer fish as model animal stems from its remarkable ability to shoot down insects settling on the foliage above the water level, and its ability to distinguish between artificial targets. Thus, the archer fish can provide the fish equivalent of a monkey or a human that can report psychophysical decisions.

## Introduction

Considerable empirical as well as theoretical effort has been devoted to investigating the neural code [1], [2], [3]. Many studies [4] have focused on coding external stimulus features according to the number of spikes fired during a time interval around stimulus onset [4], [5], [6], [7] or based on spike timing [8], [9], [10]. Exact spike timing, such as first spike latency, has also been shown to convey information about the external stimulus features in several systems, including vision [10], [11], [12], [13], [14], auditory [15], somatic-sensory [9], [16], and echolocation [17], [18], [19]. To estimate stimulus identity based on the neural responses, all of these measures require the use of an accurate stimulus onset time [2], [8], [9], [11], [14], [16], [20]. However, an internal neural representation of onset time has yet to be characterized, a fact considered by many to be a major drawback of the above coding strategy [8], [15], [16], [21]. In addition, stimulus onset time is also used implicitly by conventional rate-code readouts, e.g., the population vector [5], [22]. For cases in which neural activity represents a motor command, one may assume that an additional neural signal, in this case movement onset, encodes stimulus onset. However, in sensory systems, the onset time of the external stimulus must be deciphered from the neural responses themselves. How can stimulus onset be estimated from the noisy responses of large nerve cell populations? How accurate must the estimate of stimulus onset time be to infer stimulus identity?

Based on the framework of colour coding by the archer fish retinal ganglion cells, we investigate the representation of stimulus onset time, relying on a recent study showing that the absorption spectra of archer fish retinal photoreceptors are similar to those of humans [23]. As such, we combined behavioural and electrophysiological studies.

The outline of this paper is as follows. We start by establishing a behavioural psychophysical benchmark of the fish performance. Next we show that different readout strategies can be applied to infer stimulus colour from single cell responses, given stimulus onset time. We then show that stimulus onset time can be estimated from the population response using a relatively simple linear-nonlinear readout, and we study the accuracy of using that readout to estimate onset time. Finally, we investigate the implications of finite accuracy in onset time estimation on the ability to infer stimulus identity from single cell responses.

## Results

### Behavioural accuracy

**Figure 1:**
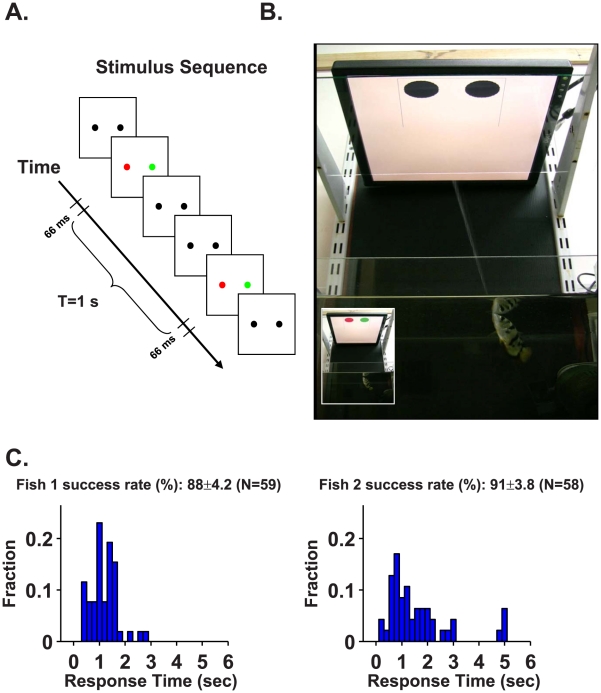
The archer fish can distinguish between two coloured targets. **A**. The stimulus sequence entails presenting the archer fish with two black circular targets. The trial began when one of the two targets was flashed red and the other green for 66 ms, after which the targets turn black for T = 1 s. The fish's task was to detect and shoot at the red target, for which it was rewarded with a food pellet (see supplementary movie S1). The targets continued to flash for 66 ms every T = 1 s with the same colour and intensity until the fish shot a target. The location (right/left) of the red target and its colour intensity were varied from trial to trial. The fish had a single attempt to shoot at each trial. **B**. Frame from a video of an archer fish shooting the left target. This is the correct selection since the left target flashed red (video frame in inset) 0.6 s before the shot. **C**. Success rates (mean ± S.E.M.) of the two fish together with response time histograms (note that the time is measured with respect to the first flash, i.e., from the moment the fish was provided with colour information). Both fish achieved very high accuracies in detecting the flashing red target. In addition, the response time histograms, which show that most of the shots were made less than 1 s after the first flash, indicate that the fish can make a decision on the basis of information from a single flash.

The utility of the archer fish as a model animal [24], [25], [26] stems from its remarkable abilities to shoot a jet of water at insects resting above the water level and to learn to distinguish between artificial targets [27], [28], [29]. Thus, the archer fish can be trained to report its psychophysical decision by shooting at its chosen target. We tested two archer fish in a behavioural two-alternative forced-choice task, during which the fish must discriminate between two coloured discs, one red and the other green, presented on a computer monitor. The animal was required to identify which of two black discs flashed red, as opposed to green, on the background of a white computer screen (Figure 1A). The location of the red disc varied randomly between trials. Since the fish “reported” its selection by shooting a jet of water at the chosen target (see Video S1 and Figure 1B), its behaviour could be used as a measure for the information content about target identity that is supplied to the brain by the retina.

Colour vision implies the ability to discriminate between variations in the spectral composition of an object irrespective of variations in intensity, and thus, the intensities of the two flashed coloured discs were varied at random in each trial. Since the intensities of the red and green discs were varied independently of each other, the fish were equally likely to encounter sessions with high red and low green lights and vice versa. To reduce the complexity introduced by fixational eye movements [see e.g.‵, 28], we used flashed targets with a duration of 66 ms (Figure 1A). During this time interval, fixational eye movement affects the responses of only 1% of the photoreceptors participating in the retinal response to the stimulus (see Materials and Methods). In addition, since body movement is an order of magnitude slower than eye movement, it can also be neglected when considering the retinal response to the dynamics of light intensities on the photoreceptor layer.

Figure 1C shows the response time histograms and colour discrimination success rates of the two archer fish. The colour discrimination success rate is about 90%. The response time histogram measures the latency period for correct shots between the presentation of the first flash and the shot. The latency for many successful shots was less than 1 s, and therefore, in those cases the psychophysical decision was based solely on the first flash of the stimulus.

The high success rate obtained in this experiment constitutes a lower bound on the ability of the archer fish retina to reliably encode information for this particular task. The small error we measured may be due to certain properties of central processing, like drifting attention or the exploration of alternative prey possibilities to investigate other potential rewards.

### Neural response to stimulus colour

To investigate the neural representation of stimulus colour that enables the high colour discrimination success rate of the archer fish, we recorded the responses of large populations of archer fish retinal ganglion cells. Stimuli of the same duration and with the same spectral properties as those used for the behavioural task were displayed on a computer monitor. As in the psychophysical experiments, the stimuli were presented at variable intensities that matched the parameters of the behavioural experiments. To avoid phase locking of the retinal dynamics due to perfect periodicity in the stimulus [30], we presented flashes with random inter-flash intervals (uniformly distributed from 1.1 s to 2.1 s). In each of these experiments, we recorded spike trains from 20–50 ganglion cells using a multi-electrode array [31], [32], [33] (192 electrodes, see Materials and Methods).

**Figure 2:**
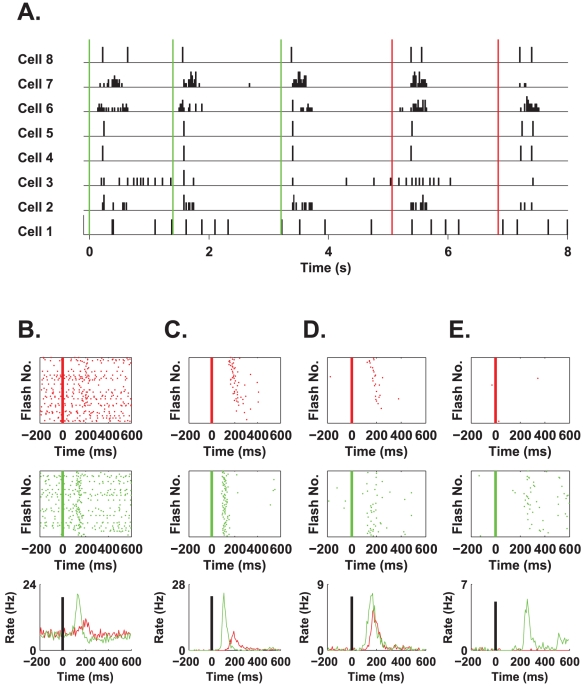
Response of the retina to flashed red/green stimuli. **A**. Normalised firing rate histograms of 8 ganglion cells in response to red and green flashes. Stimulus was displayed on a computer monitor and was matched to the temporal properties (i.e., 66 ms of coloured flash) and spectral properties (i.e., random selection of colour and intensity) of the stimulus presented to the fish during the behavioural task. The time and colour of the flashes are represented by the red or green lines from top to bottom. Note that we did not present the flashes with an exact repeat of the inter-flash interval so as to avoid strong periodicity in the retinal response and that between the flashes the screen was black as required for behavioural experiments. **B–E**. Each column represents a typical response of a single ganglion cell to flashes. Top and middle panels: raster plots in response to the red and green flashes, respectively. The flashes were sorted according to their relative intensities from low (bottom) to high (top). Response latencies varied slightly according to flash intensity. Bottom panel: time dependent mean rate of the ganglion cell response to the red and green stimuli.

**Figure 3:**
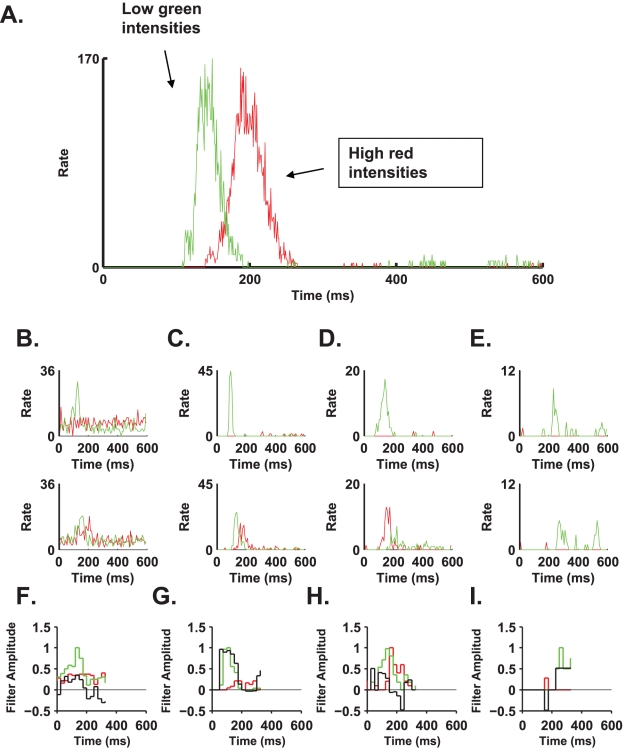
The interplay between intensity and cellular response. **A**. Here we present a PSTH of a ganglion cell, which reports on flash colour with high (90%) accuracy. The cell responded to red flashes in the intensity range of 0.55–0.65 µW/cm^2^ and to green flashes in the range of 0.22–0.32 µW/cm^2^ with a very similar response profile, although shifted in time. This is an indication that the flash onset signal is important in enabling the extraction of flash colour identity. **B–E**. The same as in A for the four cell examples in Figure 2 B–E: the top panels show PSTHs for high green intensities and low red intensities, whereas the bottom panels show PSTHs for low green intensities and high red intensities. **F–I**. The temporal filters (black) and PSTH in response to different colour flash (red and green) that were used to estimate stimulus colour using the linear-nonlinear readout, based on the responses of the four cells as shown in panels B–E of Figures 2.

Figure 2A shows the responses of eight ganglion cells to a continuous presentation of flash stimuli. Figures 2B–E shows the typical responses of four different cells, which are representative of the population, to the stimulus. In general, we found that green flashes elicited stronger responses than red flashes, with the response latency to the green flash being shorter than that to the red flash. In addition, a subgroup of cells that responded only to the green flashes was identified (Figure 2E). Typically, cells in this subgroup also exhibited a weak response to the green stimulus. The lines on the raster plots are ordered according to stimulus intensity from high intensity at the top to low intensity at the bottom (Figures 2B–E two top rows). High intensity stimuli typically resulted in stronger responses and with shorter latencies, an effect that was stronger for the red stimulus, e.g., examine red rasters (Figures 2C and D) and see also peri-stimulus time histograms (PSTHs) for different intensities of red and green stimuli (Figures 3). From the PSTHs of these cells, it is evident that stimulus identity, i.e., colour, modulates the scale, shape, and delay of the neural response (Figures 2B–E bottom row). Hence, these features can be used to estimate the stimulus colour.

### Reading out “what”

**Figure 4:**
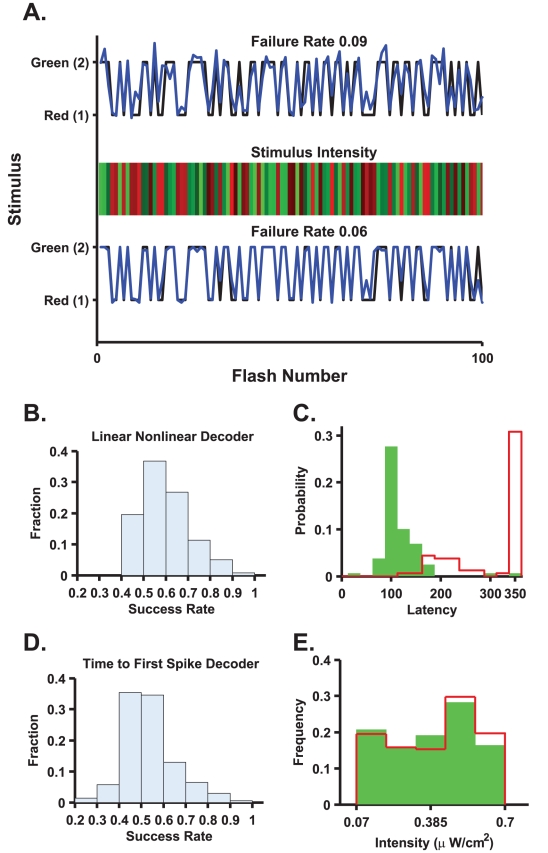
Single cells can inform the brain about flash colour given an exact time reference. **A**. Top and bottom panels represent the estimation of flash colour from two different cells (both belong to the group presented in Figure 2C). The flash colour and intensity level are depicted in the middle panel (the true intensities are apparent only on the monitor that was used for the experiments; any other monitor depicts only relative intensities); the abscissa represents the serial number of the flash. Both cells “report” the correct colour with accuracies comparable to the behavioural success rate. **B**. Histogram of success rate in detecting the flash colour of the linear-nonlinear readout of all cells from five experiments (n = 253). Note that poorly performing cells can have success rates slightly above 50% (chance-level performance) due to random fluctuations. **C**. Time to first spike distribution of the cell presented in Figure 2C. There is a clear boundary between the latency to the green flash and the latency to the red flash. **D**. Histogram of success rate of the time to first spike readout of all cells (n = 253). (The cell in Figure 2C falls within this group and the right most bin in Figure 4D.) **E**. The effect of stimulus intensity on the accuracy of colour discrimination. Errors of cells with success rates above 85% were combined (n = 17). The relative contribution of an intensity window to the overall false detection was calculated for the two colours. We did not find a strong effect of stimulus intensity on colour discrimination accuracy based on single cell responses.

We examined two readout models for stimulus identity assuming the onset time of the flash was known. The output of each of the readout mechanisms was a binary signal indicating red or green (one for red, two for green). The first readout we studied was a linear-nonlinear estimator, based on a linear filter in time (Figures 3F–I), of the neural response followed by a threshold function to decide between the two alternatives of red and green (see Material and Methods). The second readout utilized first spike time, and therefore, it was the most sensitive to response latency. Figure 4A shows two examples of stimulus colour reconstruction from the responses of two different single cells using the linear-nonlinear readout. The error rate in both examples is less than 10% (compared to the behavioural error rate of ∼10%), despite variations in the intensity of each flash. The distribution of the linear-nonlinear probability of correct discrimination across different cells in the population is presented in Figure 4B.

Figure 4C shows first spike time distribution following stimulus onset (same cell as that shown in Figure 2C) for the green (green bars) and the red (red line) stimuli. In this example, first spikes that occurred no later than approximately 170 ms after stimulus presentation resulted mainly from the presentation of green stimuli, whereas the presentation of red stimuli typically resulted in first spike times that were greater than 170 ms. Thus, stimulus colour can be estimated using first spike time relative to stimulus onset. The success rate distribution of a readout based on first spike latency showed that in both the linear-nonlinear and first spike latency readouts, ∼60% of the cells exhibited close to chance-level performance (Figure 4D). However, ∼5% of the cells were characterized by an accuracy comparable to the behavioural accuracy in both readouts (Figures 4B and 4D). Thus, given the knowledge about onset time, there exists a specialized subgroup of ∼5% of the cells that lies at the end of a continuum of less informative cells. This specialized subgroup can discriminate red from green with an accuracy comparable to that of the psychophysical accuracy. Although stimulus intensity affected the neural response (examine rasters in Figures 2B–E), we did not find that stimulus intensity strongly affected colour discrimination accuracy based on single cell responses (Figure 4E).

We further investigated whether the specialized subgroup of best cells for colour discrimination was also distinguished in other response properties. To this end, with two of the retinas we performed coloured Gaussian full-field flicker experiments in which the light intensities for each frame in the red and green channels were drawn from Gaussian distributions. We calculated the spike-triggered average of the cells and found that all the cells in the specialized subgroup were OFF cells. This bias towards OFF cells may result from two sources. First, it is possible that the archer retina has a natural bias towards OFF cells as in many amphibians [34]. Addition source for this bias may result from a measurement bias towards the OFF cells population in the extracellular recordings. For example, it was shown that slow ON cells' activity in the Salamander retina is hard to capture without special spike sorting techniques that can record the majority of cells present in a retinal patch [33], [34]. It is interesting to note that the filters that characterize the white noise response of the cells are different from the filters used for colour discrimination (data not shown). We found no other special response characteristics for the specialized subgroup of cells that performed best in the population.

To what extent is an accurate onset signal critical for the correct estimation of stimulus colour from the responses of single cells? This is best illustrated by the following example. The temporal response profiles of the cell in Figure 3A to low intensity green stimuli (green line) and high intensity red stimuli (red line) are very similar in their shape and scale, but they do not coincide; instead, the high red intensity response is displaced in time by about 70 ms. Hence, based on the cell response illustrated in Figure 3A, to discriminate low intensity green stimuli from high intensity red stimuli, knowledge about stimulus onset time with an accuracy of less than 70 ms must be used. Additional examples for the cells presented in Figures 2B–E can be found in Figures 3B–E. Of special importance is the cell presented in Figure 3C which produces ∼1 spike per flash onset albeit with different latency.

### Reading out “when”

Completion of the above readouts required that we find a mechanism that accurately estimates stimulus onset. Errors in onset estimation have two components. The first component is the detection error: the estimator may fail to signal the presence of a stimulus or may give a false alarm in the absence of a stimulus. The second is the temporal fidelity of the estimation: given a correct detection, how close in time is the estimated onset from the actual onset? This latter component can be quantified using the root mean square (RMS) value of the estimation error and by the bias that quantifies systematic errors in the onset time estimation.

**Figure 5:**
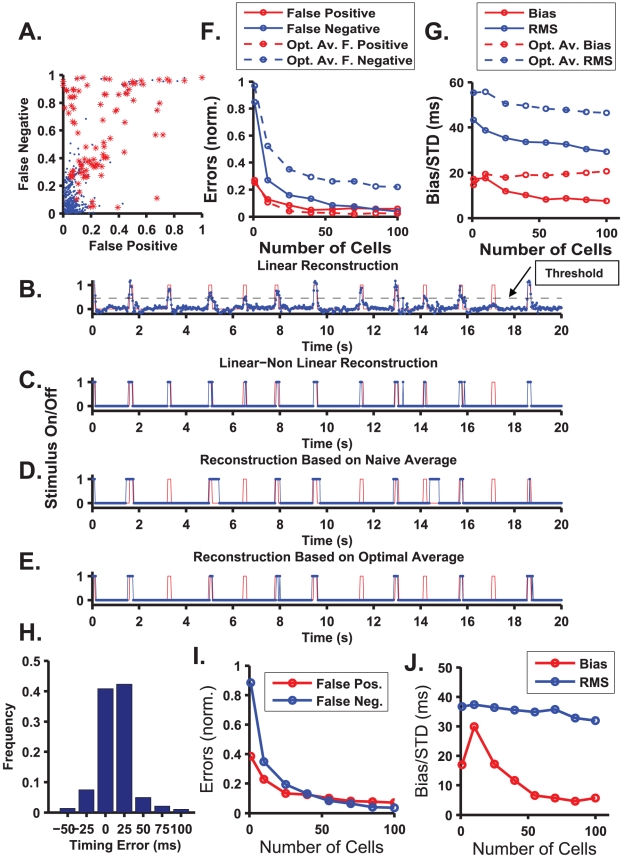
A linear-nonlinear decoder can detect the flash onset. **A**. Stimulus detection error rates using single cell (red stars) and network (blue dots) responses. Stimulus onset was estimated from the response of every single cell, using the linear-nonlinear method algorithm. The stimulus detection ability of every cell is plotted as a star in the plane of false positive and false negative error rates. Note that the false negative rate decreases with an increasing false positive rate. For every cell, the discrimination threshold was set to minimise the sum of false positive and false negative errors. False negatives were normalised by the total number of events to be detected; false positives were normalised by the total number of detected events. **B**. The linear-nonlinear stimulus onset readout mechanism. The stimulus (red line) is represented as alternating values of zero (black) and one (coloured flash). The best linear filter (blue) for estimating the stimulus from the retinal response was found (see Materials and Methods and Warland et al. [38] for details). The linear estimation was transferred through a threshold (non-linearity) to determine when the flash occurred. **C**. Comparison between the stimulus (red) and the final estimation (blue) based on the full linear non-linear estimation. **D**. Comparison between the stimulus (red) and an estimation based on a naïve average over all cells (blue). The estimation fails to reflect the stimulus properly (∼80% increase in false negatives). **E**. Comparison between the stimulus (red) and an estimation based on a weighted average over all cells (blue). While this readout outperforms the naïve average, still it is less successful then the full linear-nonlinear readout. **F**. Stimulus detection error rates as a function of the number of cells used in the linear nonlinear readout mechanism (solid line with dots) and naïve mechanism (dashed line with dots). For every population size, error rates were averaged over 50 randomly selected subgroups. Approximately 100 cells were needed to reduce both false negatives and false positives to less than 5%. **G**. Onset time estimation error, as a function of population size. Error was divided into bias and RMS error for the linear nonlinear readout (sold lines with dots) and naïve readout (dashed line with dots). In the linear nonlinear model, the bias is of order 10 ms, and it decays with the population size such that it is expected to approach zero as more cells are added. Note the decay of the RMS estimation error of the onset time to less than 30 ms. In the naïve readout, the error does not improve significantly when we add more cells. **H**. Estimated onset times histogram for a network with 85 cells. **I and J**. The same as in linear nonlinear readout of F and G but with 125 ms filters.

Examining the neural responses (Figures 2B–E), it seems plausible that single cell responses may be sufficient to estimate stimulus onset. Figure 5A shows the accuracy of a linear-nonlinear readout (see Material and Methods, reading “when”) based on single cell responses (red stars) in terms of the probability of misses (false negatives) and of the false alarm rate. Successful cells are those with low false negatives and low false positives. The absence of such cells in the bottom left corner of the plot indicates that single cell responses do not encode “when” the stimulus was presented with sufficient information to account for the psychophysical accuracy. This is in contrast to the encoding for “what” stimulus was presented, a task that single best cells perform well. Thus, the limiting factor in a single cell's ability to estimate stimulus onset is its inability to reliably *detect* the stimulus.

In order for a linear-nonlinear decoder to reliably detect stimulus onset, information must be pooled from a large population of nerve cells. We applied the linear-nonlinear readout to detect stimulus onset from a population of 100 cells selected randomly (Figure 5B–C). The stimulus (red trace) is represented as zeros (no flash) and ones (flash). A linear estimation of the stimulus (blue trace) was generated by minimizing the error between the stimulus and the estimation, after which it was passed through a threshold to obtain the decision boundary between the discrete stimulus values (blue trace, Figure 5C).

For the results presented in Figure 5, the estimated onset time was defined as follows. The output of the linear part of the estimator at time *t* (Figure 5B) is a result of multiplying the linear filter of length *T* (in Figures 5B–H we used *T* = 250 ms, in Figures 5I–J, *T* = 125 ms was used) by the neural responses from time *t* to time *t+T* (see Materials and Methods). The estimated onset time was taken to be the time *t* at which the linear part of the estimator first crossed the threshold. To estimate stimulus onset at time zero, therefore, the linear-nonlinear readout uses the neural responses up to time *T*. Onset time was considered correctly detected when the threshold was crossed 125 ms before or after the actual stimulus onset time (see onset time distribution for this example in Figure 5H). Note that the histogram decays before reaching the boundaries that we chose for defining correct detection (±125 ms). In the specific example shown in Figure 5B, the RMS estimation error of the onset time was approximately 20 ms.

The linear-nonlinear readout is a relatively simple readout, and it is widely assumed that it can be implemented by the central nervous system. The ability of the linear-nonlinear readout to detect stimulus onset serves as a proof of concept also for other, more sophisticated readout mechanisms. Naïve readout, based on the total spike count of large numbers of neurons, can also be used for stimulus detection and onset time estimation. But the naïve readout does not yield estimates of the stimulus onset that are as accurate as those of the linear-nonlinear readout. For example, Figure 5D shows readout based on the total spikes fired by all the cells in the same network followed by a selection of the stimulus value using a threshold. A central shortcoming of the naïve readout is that it does not weigh correctly noisy and informative cells. An additional estimation which is based on an optimal *weighted* average of the spike-counts of the all cells is presented in Figure 5E. As can be seen from the figure, these two naïve (rate-code) readouts suffer from larger detection errors than the linear-nonlinear readout, which takes into account the *temporal* structure of the onset response (Figure 5C).

Figures 5F and 5G show the two components of the stimulus onset estimation error (see also blue points on Figure 5A). Figure 5F depicts detection failure and false alarm rate as functions of the population size used for the estimation (see Materials and Methods). For every given population size, the error was calculated by averaging over 50 randomly chosen groups of cells. From Figure 5F, the average detection error decreases with the number of cells to a level of ∼5% false negatives and false positives for ∼100 cells. One should keep in mind that roughly 2000 ganglion cells have their receptive fields inside the target (see Materials and Methods for calculation) and hence, can provide information about flash onset to the brain. Note also that the linear non-linear readout performs better than the naïve readout (Figure 5F dashed curves). For comparison, the detection ability of the optimal naïve readout is shown (open circles). As can be seen from the Figure, although the naïve readout detection ability improves with the increase of the population size, its accuracy is considerably inferior to that of the linear-nonlinear readout, due to high false negative rate.

How accurately in time is stimulus onset estimated when the stimulus is correctly detected? Figure 5G depicts onset time estimation error as a function of the size of the population used by the linear-nonlinear readout. Error was divided into bias and RMS error. The bias represents systematic error, which is stimulus dependent, and therefore, could not be corrected with a uniform time shift that may be implemented by a delay line. Most cells exhibited longer delays in their responses to red stimuli than to green stimuli. As a result, it is expected that the mean estimation time of a red stimulus will be larger than the mean estimation time of a green stimulus. This difference is quantified by the bias that measures differences in the mean estimation times of red and green stimuli. Figure 5G (red circles) shows that the linear-nonlinear readout is biased to systematically overestimate red stimulus onset time relative to green onset time. This bias decays to zero with population size, and approximately 100 cells are required to reduce the bias below 10 ms. The blue circles in Figure 5G show the decrease in RMS estimation error of the onset time. Thus, using a population of approximately 100 cells, the linear-nonlinear readout is capable of predicting onset time with an accuracy of ∼30 ms. Surprisingly, the (optimal) naïve readout cannot overcome the bias in estimating red stimulus late, even as more cells are added to the readout (Figure 5G open circles). Thus, it appears that in order to overcome the inherent bias in estimating the onset time of a coloured target, the linear-nonlinear readout utilizes the temporal structure of the neural response.

**Figure 6:**
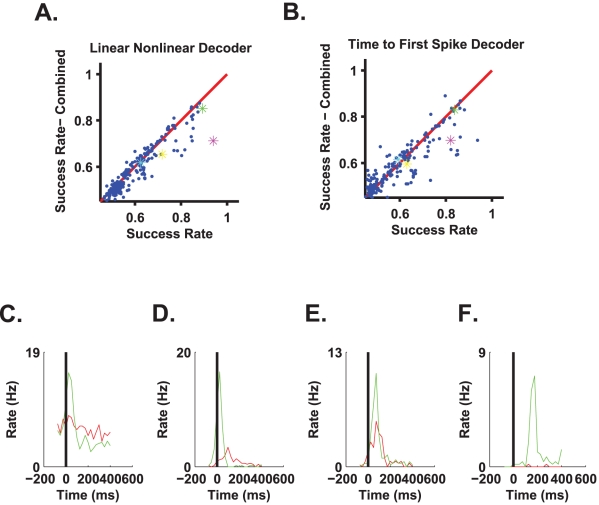
Implementation of a full two-stage decoding algorithm. In the first stage, onset time was estimated from the responses of a population of 100 cells using 125 ms filters. In the second stage, the estimated onset time was used to discriminate stimulus colour on the basis of a single cell response in a causal manner, using either the linear-nonlinear algorithm (**A**) or the first spike time code (**B**). For every cell, we plotted the accuracy of stimulus colour discrimination using the estimated onset time against its accuracy when using the exact onset time (ordinate, coloured star markers in magenta, green, yellow and cyan correspond to cells in Figures 2B, 2C, 2D and 2E, respectively.) **C–F**. Response of the four cells in Figure 2 B–E triggered by the estimated onset time from the network response. Time zero coincides with the end of the filter used for onset detection (125 ms). Thus, neural responses at positive times show the part of the response that can be used for colour discrimination by a causal readout mechanism.

Figures 6A–B show the accuracy of a combined readout that estimates both stimulus onset using a population of 100 cells and stimulus colour using single cell responses. The two stages of the readout—onset detection and colour discrimination—were combined in a causal way. The onset detector used the neural responses during a time interval of *T* = 125 ms (see Figures 5I, J for onset detection accuracy using this time interval), and the colour discriminator used only spikes that were fired after that time interval. Thus, the spikes used for reading out “when” were not used for reading out “what”.

In the combined readout (Figures 6A–B) we re-calculated the optimized filters for reading out “what”, using two approaches. *i*) The colour discrimination weights were learnt using the accurate onset time with a time shift of length of the filter used for the onset detection (see Material and Methods). *ii*) The parameters of the colour discrimination were learnt using the statistics of the neural responses following the estimated onset time. The results of Figure 6 were obtained using the first approach, i.e., training the colour discriminator using the exact time. Using the second approach, i.e., training the colour discriminator using the estimated onset time, added more noise to the learning process and yielded a somewhat inferior readout due to the finite data set (results not shown).

Figures 6C–F show the PSTHs of the cells presented in Figures 2B–E with time zero as given by a causal onset detector. The main difference between PSTHs computed with the estimated onset signal and PSTHs computed using the actual stimulus onset time is in the latency of the response. The accuracy of the combined readout is compared to the accuracy of a readout that used the exact stimulus onset time (e.g., Figures 4B and 4D). As can be seen, although performance slightly deteriorates when stimulus onset time is estimated from the neural responses (most points are below the red identity line), this deterioration is in many cases small. Note that in contrast to the common belief, the time to first spike decoder does not appear to be considerably more sensitive to errors in onset time estimation than the linear-nonlinear decoder. The capacity of the first-spike decoder to perform well in the two stage readout results from the ability of the linear non-linear onset detector to overcome the bias and to obtain standard error that is typically smaller than the latency difference to red and green stimuli of tuned cells.

## Discussion

The task confronted by the archer fish retina is far more demanding in a natural environment, where it must calculate the visual object's location and whether the object is in motion, than in the experimental situation described here. The experiment was designed to evaluate the difficulties associated with deciphering an event in continuous time. As such, we used a simplified, two-alternative forced-choice visual discrimination task. Our work presents a two-stage readout mechanism capable of estimating stimulus colour in continuous time (i.e., when the stimulus is presented and without a cue for or prior knowledge of its onset) with an accuracy comparable to psychophysical accuracy.

The first readout stage estimates stimulus onset time. We found that due to the *rarity* of a stimulus presence event, a population of approximately 100 cells was required to estimate onset time with sufficient accuracy. This requirement does not result from the need to decrease the noise in the estimated time, but rather from the need to reduce detection failure and false alarm rates. In the second stage, the estimated onset time calculated in the first stage is used to extract information about stimulus colour from the rich temporal structure of single cell responses. We found that approximately 5% of the cells can be considered colour specialized cells that encode information at a level of accuracy comparable to psychophysical accuracy. It was shown that this information can be extracted using either linear-nonlinear or first spike time readouts, with similar performance.

### Additional readout mechanism

Here we suggested a two stage readout mechanism that separates the coding for the time of stimulus appearance from the coding of stimulus identity. This separation may not be essential, as other readout mechanisms are possible and stimulus appearance and identity may be decoded from the neural responses using a single stage readout mechanism. The utility of separating the coding for “what” from that for “when” is that it highlights the difficulty in detecting stimulus onset as opposed to distinguishing its colour, which is easier. We have shown that stimulus detection may be achieved using a relatively simple mechanism, a linear-nonlinear readout, albeit based on the responses of large nerve cell populations. Can response latency also serve as a cue for stimulus detection, and if so, how can it be used without a prior estimate of stimulus onset?

**Figure 7:**
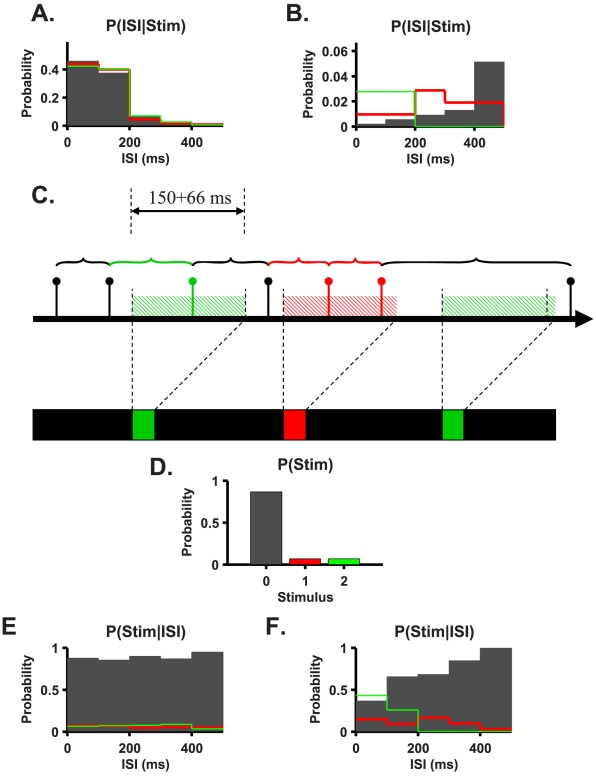
ISI based decoder. **A** and **B**. The conditional probability of ISIs given the stimulus for cells presented in Figures 2C and 2D. **C**. Reconstruction of the joint probability distribution of the stimulus and ISIs was done by first generating the ISI time series and assigning each spike the ISI with the previous spike. If the spike fell within 216 ms after the flash onset (150 ms+66 ms), it was assigned to the colour of the flash. Otherwise, it was assigned to black (no flash). The joint probability distribution was generated by scanning all the stimulus-ISI (response) pairs. **D**. Stimulus prior distribution. Note that this probability distribution is controlled by the experimentalist and hence is arbitrary and does not reflect neuronal coding properties. **E** and **F**. The posterior distribution of the presence of stimuli given the ISIs for cells presented in Figures 2C–D.

Lacking knowledge about stimulus onset, one cannot determine absolute spike time latency; rather, one may only use the relative latencies to estimate the time of stimulus appearance [10], [12]. Relative spike time latencies of a single cell are simply the inter spike intervals (ISIs) of that cell. Figures 7A and B show the ISI distribution of two single cells for the three cases comprising red stimulus, green stimulus, and black screen. Calculation of the joint ISI and stimulus histograms is illustrated in Figure 7C. Every ISI was linked to a stimulus condition by the time of its second spike. If there was a green or a red stimulus during the 150 ms preceding the previous spike, the ISI was assigned to that stimulus. Otherwise, the ISI was assigned to the black screen stimulus. We varied the 150-ms time window with no significant change in the results. This procedure provided us with the ISI distribution that was conditioned on stimulus colour.

The *maximum likelihood* estimator chooses the stimulus that maximizes the likelihood of a response. The algorithm requires that if we observe a certain ISI, we need to select the stimulus value with the maximum probability to observe this specific interval. Thus, examining the responses in Figure 7A, relatively short ISIs of less than 400 ms and more than 100 ms will be classified by the maximum likelihood estimator as originating either from a red or a green stimulus. Given a black screen, false alarms will result from ISIs in the range of 100–400 ms. In this case, about 50% of the ISIs in response to a black screen will generate a false alarm. Since the baseline firing rate of this cell is about 6 Hz, the maximum likelihood detector will generate a false alarm about every 300 ms of black screen, on average. Even if the overlap between the conditional distributions was smaller, for example only 10% of the ISIs, the maximum likelihood detector would generate a false alarm every 1.5 s, on average. The high false alarm rate of the maximum likelihood results in part from the black screen's being a common stimulus that is present most of the time. Moreover, the maximum likelihood ignores prior knowledge of stimulus distribution: stimulus presence (red or green) is a rare event (Figure 7D).

The *maximum a posteriori* estimator takes prior information about the stimulus into account. Figures 7 E and 7F show the posterior distribution of instances of stimulus onset for different ISIs. Essentially, the relative value of every bar in the histogram of the conditional probability (Figures 7E and 7F) was multiplied by the ratio of prior probabilities of the stimulus (and normalized by the marginal probability of the ISI), thereby substantially decreasing the posterior probability of a rare event. Taking into account prior knowledge of stimulus occurrence (Figure 7D), the maximum a posteriori readout will almost always estimate a black screen. Thus, ignoring prior knowledge about the frequency of a rare event yields a high degree of false alarms; taking that knowledge into account, however, results in a high degree of false negatives.

Alternatively, one could expect that using cells with low spontaneous firing rates—for example, the cell of Figure 7B—will help to decrease the false alarm rate. However, these cells are also characterized by a very weak response that is typically at most one spike per stimulus. Hence, the ISI distribution (Figure 7F) does not reflect the stimulus response and yields poor detection power. To overcome the high level of error rates, information about presence of stimuli needs to be pooled from the responses of a relatively large population of cells. In addition, one should bear in mind that a readout that decides according to the relative spike times of a cell that fires at a baseline level of 10 Hz may yield a different estimate on an average of ten times a second. Thus, relative latencies do not seem to solve the problem of stimulus onset detection.

In a recent work, Gollisch and Meister [10] suggested using the relative timings of the spikes from two different cells to circumvent the problem of unknown onset. Above we analysed a somewhat different but similar scenario of the ISIs of a single cell response. Nevertheless, as in the above example, one expects that an estimator based on the relative latencies of two retinal ganglion cells firing at a spontaneous rate of about 10 Hz will result in a high degree of errors. As above, the difficulty here results from the fact that stimulus onset is a rare event in time.

Previously, Chase and Young [15] investigated first spike time latency code in the auditory system of the cat. Using mutual information, they concluded that estimating an onset signal from a pseudopopulation (a population that is composed of single cells that were recorded separately) does not decrease, and on average slightly increases, the information content embedded in the first spike time latency of single cells. Nevertheless, it remained unclear whether this amount of information is sufficient to account for the psychophysical accuracy of the animal. This issue has been addressed in our study.

### Colour vision in the archer fish

Traditionally, three standard methods are used to demonstrate colour vision in animals [35]. The first method entails finding an isoluminance point, i.e., a point where two test monochromatic lights are perceived to be of equal luminance. Identifying an isoluminance point in animals, however, is not a trivial task.

The second way to demonstrate colour vision is to vary the intensity over a considerable range, typically over three to four log scales [35]. This is the basic paradigm that we used here, albeit in a limited manner, i.e., testing only red vs. green targets with 1 log unit intensity variations. The intensity ranges in our experiments were dictated by the limitations of the computer monitors that served as targets for the archer fish. In addition, we decided to use flash coloured targets to avoid the complexity involved in eye movement effects. Due to the short duration of the flash and the need to use a flexible display monitor that is later used for electrophysiology with a multi-electrode array, we limited the variations in intensity to 10 fold (1 log unit) between the lowest and highest intensities used in the experiment. Additionally, we searched for patterns of errors in the psychophysical task. If the archer fish does not see colour and discriminates based on their perceived brightness—for example, red may be perceived darker than a green of the same luminance—then one would expect the errors to be correlated with the relative intensities of the coloured stimuli. We did not find such a correlation structure in the psychophysical data, implying that stimulus intensity is not the basis for the psychophysical errors (see also Figure 3E for the distribution of colour discrimination errors based on single cell responses).

The third approach is the “gray card” experiment developed by von-Frisch. In this paradigm, the animal has to select a coloured target embedded in an array of gray destructors [36]. Thus, we further tested a red trained fish with a red target against grey card targets, i.e., an ensemble of six gray targets with different intensities. The success rates were 93% and 95% for two fish (N = 30 and N = 24, respectively) at a chance level of 16%.

It is interesting to note that in a recent report, Temple et al. [23] demonstrated that the cone distribution in the archer fish retina varies across the retina in a way that matches the different visual environments, i.e., aquatic and areal, confronting the archer fish visual system. This is an additional indication that archer fish may possess colour vision.

Although our results support the hypothesis that archer fish have colour vision, further work is required to fully test this claim. Nevertheless, the aim of the current study was not to investigate whether archer fish have colour vision, but rather to utilize this framework to study the problem of estimating stimulus onset from the neural response.

## Materials & Methods

### Ethics statement

All experiments with fish were in accordance with Ben-Gurion University of the Negev regulations and government regulations.

### Training the archer fish to shoot at a target presented on a computer monitor

The experimental setup consisted of a computer monitor (Dell 1708FP flat panel LCD monitor) situated 30 cm above the water level of a fish tank facing towards the water. A glass plate was fixed over the screen to protect the monitor. The monitor was connected to a laptop, and training sessions were generated in a slide-show manner using the PowerPoint program. The emission spectra of the red and green monitor channels were characterized by a narrow band of emission. The training and experimental procedures were conducted separately for each of the two naive archer fish (*Toxotes chatareus*). The training has started by presenting the fish with a picture of an insect and rewarding a hit with a food pellet. Experiments were limited to five-day periods, with two days of rest in between so as to minimize the risk of overfeeding.

The behavioural task was designed to test the ability of the fish to discriminate between the different colours. In each session, two circular targets 4.5 cm in diameter situated on a white background were flashed for 66 ms from black to green or red and back to black once a second (8-bit colour image) with a randomized location (see Video S1, Figures 1A and 1B). Since the archer fish eye moves by less than the diameter of a single photoreceptor during the 66 ms flash, the contribution of eye movements to retinal encoding in this task could be neglected. Following Jacobs et al. [37], red and green disc intensities were selected randomly and independently at values between 0.07–0.7 µW/cm^2^. The fish was trained to shoot at the red disc by rewarding it with food when it shot the correct (red) target. To determine whether the fish had indeed hit the target, we examined the pattern of water created by the water jet from the fish on the glass plate. The place the jet touched the monitor was indicated by a water droplet on the monitor corresponding to the centre of the jet. A hit was easy to detect since the spacing between the discs was very large compared to the size of the water droplet on the monitor. Each fish required one to two months of training before reaching its best performance. The training sessions were filmed with a digital video camera (Sony Handycam DCR-HC23, 25 frames per second) for later analysis of fish response time to chromatic stimulus.

### Computer monitor radiometric calibration

The spectral output of the computer monitor was measured with a Red Tide USB650 CCD spectrometer (Ocean Optics, Dunedin, Florida, USA). The spectrometer was calibrated to absolute radiometric units by using a LS-1-CAL calibrated tungsten halogen lamp (Ocean Optics, Dunedin, Florida, USA).

### Electrophysiology of retinal ganglion cells

Archer fish retinas were isolated from the eye in the dark after a period of 1 h of light adaptation. Experiments were performed at noontime. Each retina was peeled from the sclera together with the pigment epithelium and placed in a petri dish with a glass bottom, with the ganglion cell layer facing down. Retinas were superfused with oxygenated (97% O_2_/3% CO_2_) Ringer's medium [28] at room temperature. A 192 fakir-bed-like multi-electrode array was produced by placing two Cyberkinetics 3D multi-electrode arrays side by side (Cyberkinetics, Salt Lake City, Utah, USA). The array was lowered onto the retina from above by means of a standard mechanical manipulator. Extracellularly recorded signals were digitized at 10 kSamples/s on four PCs and stored for off-line analysis. Spike sorting was done by extracting from each potential waveform amplitude and width, followed by manual clustering using an in-house written MATLAB program. Data from five retinas taken from three different animals are presented (total number of cells used 253). The low yield of cells from each experiment was due to the fact that the retina is not flat and therefore only part of the array captures spikes from ganglion cells.

### Stimulus

The stimulus for *in vitro* retinal preparation was presented on the same LCD monitor used in the behavioural experiments. To mimic the visual information flowing to the retina during the behavioural experiments, we used a full field stimulus. Since the size of the disc on the retina during the behavioural experiments was ∼600 µm and the ganglion cell receptive field radius is ∼100 µm (as measured with a random checkerboard [28]), we made the approximation that each cell “sees” a full field flash. The stimulus generated using the LCD computer monitor consisted of multiple red or green coloured flashes matched to the behavioural experiments in time sequence (66 ms flash time) and spectral properties (i.e., random selection of colour and intensity).

### Training the linear-nonlinear decoder for colour discrimination (“what”)

The linear-nonlinear decoder was based on linear estimation followed by a threshold function. We started by representing the stimulus colour of the 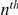 trial 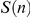 with 1 for red and 2 for green. For the linear estimation, we followed Warland et al. [38] and represented the response of a neuron with a rate function with overlapping windows of 25 ms (50% overlap). Let 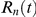 be the number of spikes generated by the neuron at time window *t* at the *n* trial and 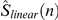 the linear stimulus estimation. The estimation of the stimulus was obtained from the ganglion cell responses by taking the dot product of the response 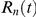 with a linear filter. Specifically we obtained:
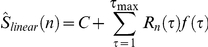
where 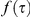 is the linear filter at time *τ* before the current time bin, *C* is a constant, and 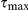 is the filter length (time zero refers to stimulus onset or estimated onset in the combined readout). The filter 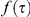 was obtained by minimising the square error between the stimulus and the estimation, i.e., we chose 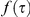 such that 
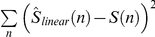
 was minimised. Then we passed 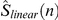 through a nonlinearity 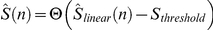, where 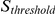 was the threshold. The threshold was selected such that it minimised the error between the stimulus and 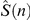 using a standard MATLAB toolbox (MathWorks Inc.). The length of the filter was 375 ms. The value of the optimal threshold constant, *C*, is very close to 1.5.

For the purpose of the combined readout, i.e., when we used the network signal as a time reference (Figure 6), we re-calculated the optimal linear filters for red/green discrimination in two methods. *i*) Parallel learning: the accurate onset time with a time shift of length of the filter used for the onset detection were used. *ii*) Serial learning: the parameters of the colour discrimination were learnt using the statistics of the neural responses following the estimated onset time. This is done by first learning the onset detector parameters. Then we apply the onset detector to find the onset timing stamps on the same training data set. Finally, using these time stamps we learn the colour discriminator weights

Although the algorithm presented here is only one approach for finding the linear-nonlinear decoder and it has no guarantee of optimality, for our purposes it was sufficient as it allowed us to demonstrate that the information indeed existed in the population. The data was cross validated by dividing it into two segments and then learning the decoder parameters on one segment and evaluating the decoder success on the second.

### Training the linear-nonlinear decoder for onset detection (“when”)

Following Warland et al. [38] we represented the response of each neuron with a rate function with overlapping windows of 25 ms (50% overlap). Let 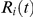 be the number of spikes generated by the *i^th^* neuron at time window *t* and 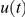 the linear stimulus estimation at the *t^th^* time interval. Stimulus estimation was obtained from the ganglion cell responses by convolving the response 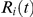 with a linear filter. Specifically we get:
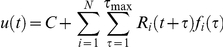
Where *N* is the number of cells, 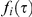 is the linear filter for the *i^th^* cell at time *τ* before the current time bin, *C* is a constant, and 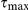 is the filter length. The filter 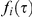 was obtained by minimising the square error between the stimulus and the estimation, i.e., we chose 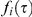 such that 
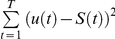
 was minimised using a standard MATLAB toolbox (MathWorks Inc.). Then we passed 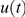 through a nonlinearity 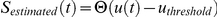, where 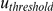 is the threshold. The threshold was selected again such that it minimised the error between the stimulus, i.e., we required that 
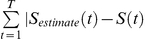
 was minimal. In this way the false hit and false detections were treated equally. The data was cross validated by dividing it into two segments and then learning the decoder parameters on one segment and evaluating the decoder success on the second.

Error was calculated as follows: a false positive was defined as a detected flash that was more than 125 ms from the nearest true flash. A false negative was defined as a true flash for which there was not a detected flash less than 125 ms away. The timing errors were calculated only on the detected flashes. Since we required that the maximal detected flash be no more than 125 ms away from the true flash, there was a bound on the timing error.

Two naïve rate code onset detectors were studied. The first was based on the population rate, i.e., the total spike count in the entire population during a predefined time interval (Figure 5D). In the second naïve readout we allowed an optimal selection of the weight for each cell, i.e., the decision was based on a weighted average of the spike counts of different cells with optimal weights (Figure 5E). Hence, the more noisy cells were assigned lower weights in the decision process. Specifically, for the results presented in Figure 5 we have used 250 ms. Additional time intervals were also studied with no significant improvement in the detection performance.

### Time to first spike decoder

The time to first spike decoder was constructed by first estimating the joint probability of time to first spike and stimulus identity on a training data set. We then used a maximum likelihood decoder to estimate the stimulus on a test data set.

### Estimating the fraction of photoreceptors affected by eye movements during flash stimulus

Simple geometrical considerations yield that a target of size 4.5 cm located at a distance of 30 cm from an eye of diameter 4 mm spans roughly 0.60 mm on the retina. Given the photoreceptor diameter of 6 µm, about 7,000 photoreceptors will be stimulated by the flash stimulus. The fixational eye movement oscillation period of the archer fish [28] is approximately 200 ms, during which the image on the retina shifts at about 6 µm, which is about one photoreceptor diameter. Hence, during 66 ms of the flash stimulus, an order of magnitude of only ten photoreceptors will be affected due to fixational eye movements.

### Estimating the number of ganglion cells participating in encoding flash onset

We used the following parameters: density of 4,500 cells/mm^2^, receptive field radius of ∼100 µm, and eye radius of 4 mm [28], [39]. We assumed that cells that encode information have their receptive fields at least partially covering the target, resulting in an estimate of 1,000–2,000 cells that participate in the encoding process. This is, of course, a lower boundary since we took into consideration only receptive field centres.

## Supporting Information

Video S1 Archer shoot target. Two successful shots of the archer fish in a behavioural experiment. In the first (second) shot the red flash was to the left (right). The fish shoots the target (faint white jet) and the experimentalist rewards it by placing a food pellet in the water tank. A clear view of the event sequence during the experiment can be obtained by viewing the frames one by one.
(6.96 MB MOV)
